# Designing Formulation Strategies for Enhanced Stability of Therapeutic Peptides in Aqueous Solutions: A Review

**DOI:** 10.3390/pharmaceutics15030935

**Published:** 2023-03-14

**Authors:** Primawan Putra Nugrahadi, Wouter L. J. Hinrichs, Henderik W. Frijlink, Christian Schöneich, Christina Avanti

**Affiliations:** 1 Department of Pharmaceutics, Faculty of Pharmacy, University of Surabaya, Jalan Raya Kalirungkut, Surabaya 60293, Indonesia; 2 Department of Pharmaceutical Technology and Biopharmacy, University of Groningen, 9701 BA Groningen, The Netherlands; 3 Department of Pharmaceutical Chemistry, School of Pharmacy, University of Kansas, 2095 Constant Avenue, Lawrence, KS 66047, USA

**Keywords:** therapeutic peptides, stabilization formulations, aqueous solutions

## Abstract

Over the past few decades, there has been a tremendous increase in the utilization of therapeutic peptides. Therapeutic peptides are usually administered via the parenteral route, requiring an aqueous formulation. Unfortunately, peptides are often unstable in aqueous solutions, affecting stability and bioactivity. Although a stable and dry formulation for reconstitution might be designed, from a pharmaco-economic and practical convenience point of view, a peptide formulation in an aqueous liquid form is preferred. Designing formulation strategies that optimize peptide stability may improve bioavailability and increase therapeutic efficacy. This literature review provides an overview of various degradation pathways and formulation strategies to stabilize therapeutic peptides in aqueous solutions. First, we introduce the major peptide stability issues in liquid formulations and the degradation mechanisms. Then, we present a variety of known strategies to inhibit or slow down peptide degradation. Overall, the most practical approaches to peptide stabilization are pH optimization and selecting the appropriate type of buffer. Other practical strategies to reduce peptide degradation rates in solution are the application of co-solvency, air exclusion, viscosity enhancement, PEGylation, and using polyol excipients.

## 1. Introduction

**Figure 1:**
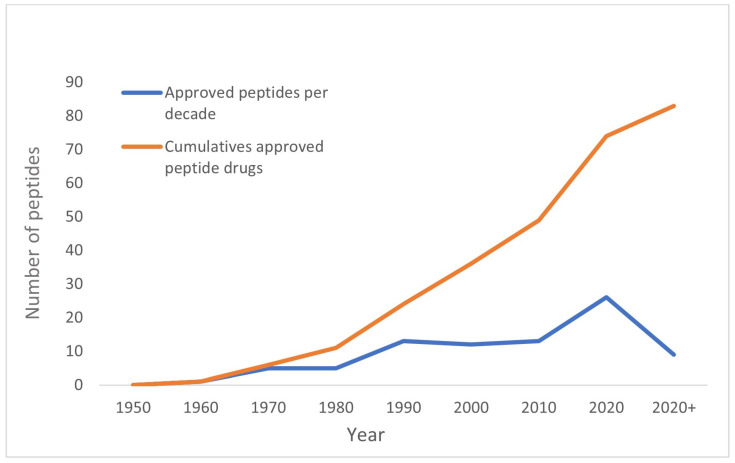
Increasing number of approved peptides in the United States, Europe, and Japan [3,4].

Advances in biotechnology have resulted in an increasing number of therapeutically active peptides entering the market. Since du Vigneaud [1] successfully synthesized oxytocin in 1953, there have been significant achievements in discovering peptides as active pharmaceutical ingredients in the following decades. Currently, over 80 peptides have been approved as therapeutic agents in the United States, Europe, and Japan, as shown in Figure 1 and Table 1. Additionally, more than 160 peptides are undergoing clinical trials, and more than 200 are in the preclinical stage [2]. This tendency is predicted to persist in the future. Table 2 shows peptide drug candidates currently in a clinical trial [3].

Peptides can control various physiological processes, functioning as growth factors, neurotransmitters, and endocrine or paracrine signals at other sites of action. In diverse disease areas, such as endocrinology, oncology, hematology, and urology, peptides are used as therapeutic agents [4]. Several antibiotics, antitumor agents, hormones, and neurotransmitters are peptides.

Peptides are different from proteins. Although both are composed of amino acids, peptides are smaller molecules comprised of two or more amino acids linked by peptide bonds, while proteins are long chains of amino acids that may have a much larger number of amino acids. Unlike proteins with a defined tertiary and quaternary structure [5], peptides generally do not have a defined three-dimensional structure. Although peptides are mostly linear and usually do not have as much complexity in their structure as proteins, some can have a defined three-dimensional structure due to the presence of multiple disulfide bridges, hydrogen bonds, and hydrophobic interactions [6,7]. The hydrophobic sides of amino acids in peptides are buried inside their structure and tend to form aggregates. This is because hydrophobic, non-covalent interactions between non-polar or slightly polar molecules cause these side chains to avoid contact with water and interact instead. This tendency to aggregate can also be increased by changes in pH, temperature, ionic strength, and the presence of surfactants or other excipients [8]. Furthermore, their functionality in living organisms is different. While proteins usually act as structural and regulatory molecules [9], peptides regulate a broad spectrum of biological effects, including proteins [10,11]. Making a clear distinction between peptides and proteins based on the number of amino acids is challenging, and several definitions exist. First, the United States Food and Drug Administration defines peptides as short chains that contain less than 40 amino acid residues [12]. Malavolta [13] provides a similar definition, defining molecules containing 50 amino acid residues or more as proteins. Between them is a category called polypeptides that have 40–49 residues. Furthermore, Forbes [14] defines peptides as a short string of 2 to 50 amino acids, where oligopeptides contain between 10 and 20, and polypeptides contain more than 20 amino acids. Our review will focus on therapeutic peptides composed of fewer than 50 amino acids.

Therapeutic peptides have many challenges regarding their formulation and administration. Peptides are often sensitive to digestive enzymes and have a limited ability to permeate intestinal membranes, leading to poor bioavailability after oral administration [15]. Furthermore, peptides are prone to chemical and physical instability, which may cause them to degrade during preparation, manufacturing, and storage.

The poor oral bioavailability of peptides has encouraged the exploration of alternative non-invasive delivery methods of peptides, such as buccal [16,17], vaginal [18], ocular [19], percutaneous [20], rectal [21], nasal [22], transdermal [23], and pulmonary [24] routes. Although non-invasive delivery routes for peptides have been continuously developed, they have failed to produce satisfactory outcomes when a rapid onset is required. Consequently, the parenteral route remains the predominant method for administering therapeutic peptides. Intravenous injection is the most direct route for delivering peptides into the systemic circulation, providing immediate and complete bioavailability. Some peptides are administered intramuscularly, injected directly into a muscle, absorbed into the bloodstream, and distributed throughout the body. Both intramuscular and intravenous routes are not accessible to self-performed administration, and patients experience pain and discomfort after the injection. The subcutaneous route can show a peak level within 30 min [25]. This route can be employed and is more suitable for self-administration.

Due to their potential instability, most peptide drugs require storage and transportation at low temperatures, also referred to as the cold chain. The availability of therapeutic peptides is significantly impacted by this instability, especially in tropical and remote areas where a cold chain is unavailable [26,27]. An urgent strategy is required to address peptide instability, particularly in an aqueous solution for injection, which is favored compared to lyophilized powder. Despite lyophilization using appropriate stabilizing, and excipients appearing to be an ideal approach in maintaining the integrity of peptides [28], it is unfortunately time-consuming and costly from an economic standpoint [29]. Furthermore, lyophilized products may be too expensive for developing countries. Reconstitution also poses a risk of contamination [30]. The volume and mass of freeze-dried products, including both vials used for lyophilized powder and its reconstitution liquid, are typically up to twice the size of those used for liquid formulations, resulting in more extensive packaging material, larger storage area, and higher transportation costs [29]. Finally, reconstituting the dried product may be inconvenient and difficult for patients. Therefore, liquid formulations are preferred if they are sufficiently stable.

Peptide stability in aqueous solutions is a critical aspect when developing parenteral formulations, as the potency of a peptide is often compromised due to chemical or physical degradation pathways [31]. Having a comprehensive understanding of the underlying instability mechanism of a particular peptide is crucial to optimizing its stability in the final formulation during the pharmaceutical development process [28]. The aim of this review is to explore different degradation pathways of peptides and to propose several rational strategies (excluding chemical modification of the peptide) for improving the stability of therapeutic peptides in aqueous solutions.

## 2. Instability of Peptide and the Possible Causes of Degradation

Peptides may be able to undergo several degradation pathways. Peptide degradation can occur through chemical and physical mechanisms. Chemical instability involves processes that alter the peptide by creating or breaking covalent bonds, leading to the formation of new chemical entities [32]. Oxidation, hydrolysis, β-elimination, deamidation, racemization, isomerization, and disulfide exchange are examples of chemical instability pathways [33,34]. Physical instability refers to structural changes in non-covalent interactions of the peptides and includes changes in secondary structure, adsorption, aggregation, and precipitation [28]. Table 3 shows various degradation pathways of peptides in an aqueous solution and influencing parameters.

### 2.1. Hydrolytic Pathways

#### 2.1.1. Chain Cleavage of the Peptide Backbone

Hydrolysis represents one of the main degradation pathways of peptides. Generally, hydrolysis is catalyzed by Bronsted acids and bases [61] and strongly depends on the pH. This pH dependency has been extensively investigated for the peptides gonadorelin and triptorelin. These peptides undergo acid-catalyzed hydrolysis at pH 1–3 through deamidation of the C-terminal amide. At pH 5–6, however, the peptide backbone can undergo hydrolysis at the N-terminal side of the serine (Ser) residue. This process is likely facilitated by the hydroxyl group on the Ser side chain, which acts as a nucleophile by attacking the adjacent amide bond. As a result of this reaction, a cyclic intermediate is formed, which ultimately leads to the fragmentation of the peptide [62,63]. At pH > 7, the primary degradation pathway of gonadorelin and triptorelin are base-catalyzed epimerization. The epimerization reaction most likely involves Ser via a carbanion intermediate. Gonadorelin and triptorelin have the capability to create hydrogen bridges in a relatively stable six-membered intermediate, which elucidates the reason for the Ser residue’s relatively high rate of racemization in comparison to other amino acids. Apart from epimerization, the hydrolysis of gonadorelin and triptorelin under base-catalyzed conditions has also been detected [62,64,65]. Recombinant Glucagon-like Peptide-1 (r-GLP-1) has also been reported to undergo base-catalyzed racemization because of extreme pH exposure during purification that can impact its impurity profile and yield of bulk rGLP-1 [66]. The primary degradation route of recombinant human parathyroid hormone (rhPTH) occurs via cleavage at the aspartate (Asp) residue under acidic conditions. Conversely, when the pH is above 5, asparagine (Asn) deamidation is the primary degradation route [67]. The cholecystokinin peptide tends to undergo C-terminal and N-terminal cleavage as the primary degradation pathways when it is subjected to non-isothermal conditions [68].

Somatostatin and its analog octastatin have also been observed to undergo acid/base-catalyzed hydrolysis in aqueous formulations, with the rate of hydrolysis being influenced by the buffer species [69,70]. Octastatin, for example, experiences a higher degradation rate in a phosphate buffer than in a glutamate buffer solution, likely due to a catalytic effect of phosphate ions [69]. It appears that increasing phosphate concentration results in much faster degradation of octastatin. Conversely, increasing the concentration of glutamate in a buffer solution enhances the stability of the solution, as evidenced by hydrophobic and ionic interactions between glutamate and octastatin [67]. These findings underscore the significance of selecting appropriate buffer species and their concentrations when formulating peptides.

#### 2.1.2. Deamidation of Asn and Gln Residues

Peptides containing glutamine (Gln) and Asn residues are susceptible to deamidation, leading to the formation of Glu and Asp, respectively, under physiological conditions. When the pH is lower than 3, Asn residues deamidation occurs primarily through the direct hydrolysis of the Asn amide side chain to generate Asp. Likewise, Gln residues undergo acid-catalyzed direct hydrolysis to form Glu [32]. Asn deamidation mostly transpires via a cyclic imide intermediate that forms through an intramolecular reaction where the amino acid residue’s nitrogen next to Asn attacks the carbonyl carbon on the side chain of the Asn residue. Thus, the rate of deamidation through this pathway depends on the carboxyl-side amino acid residue’s nature [71,72,73]. Under similar conditions, the deamidation of Gln residues proceeds much slower than the deamidation of Asn, because the cyclization of Asn residues into a five-membered ring is kinetically more favorable than the formation of a six-membered ring intermediate in Gln deamidation [32].

Peptide chain flexibility strongly favors a high rate of Asn deamidation [74]. The amino acid sequence in the peptides can also affect the rate of deamidation [75]. Amino acid residues following Asn, such as threonine (Thr), Ser, and Asp, may substantially increase the reaction rate since they are very susceptible to dehydration, forming a cyclic imide intermediate [8].

At alkaline and neutral pH, adrenocorticotropic hormone (ACTH), was shown to degrade via deamidation of its single Asn residue [71,76]. Asn or Gln deamidation was also observed for salmon calcitonin (sCT) under acidic conditions [77]. Oxytocin provides another instance of a peptide that can be subjected to Asn [78] and Gln [79] side chain amides deamidation through hydrolysis. Additionally, oxytocin’s C-terminal glycine (Gly)-NH has been reported to undergo deamidation at pH 2 [26].

#### 2.1.3. Isomerization of Asp Residues

**Figure 2:**
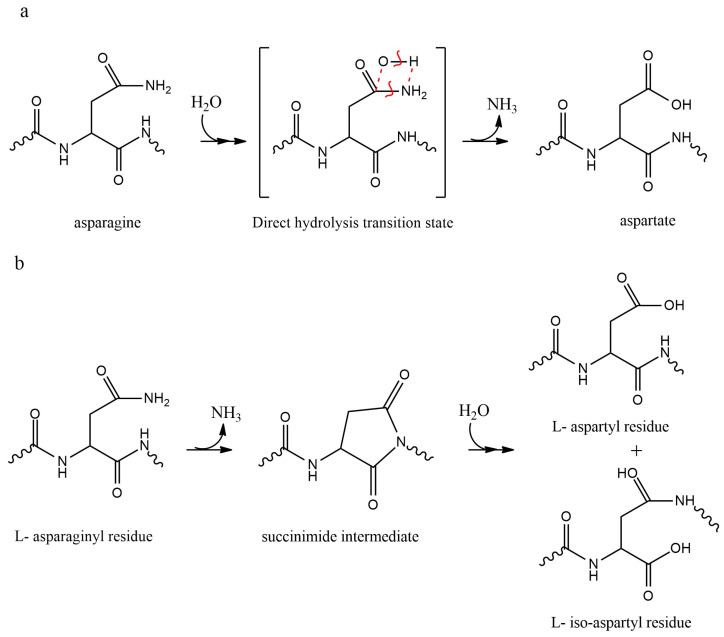
Deamidation pathways of asparagine through (**a**) direct hydrolysis and (**b**) succinimide mediation [85]. Red lines show the proton transfer from water molecule to the leaving group (-NH_2_).

The Asp transformation into isoAsp follows the equivalent succinimide ring intermediate as reported for Asn deamidation [80,81] (see Figure 2). Moreover, racemization of L-succinimide into D-succinimide can produce D-Asp and D-isoAsp enantiomers [72,82]. The rate-limiting step for the isomerization of Asp and Asn deamidation reactions at physiological pH is the formation of the succinimide intermediate [83]. Isomerization of the Asp-hexapeptide into the isoAsp-hexapeptide through cyclic imide intermediate was also reported to be pH dependent [84].

### 2.2. Oxidative Pathways

**Figure 3:**
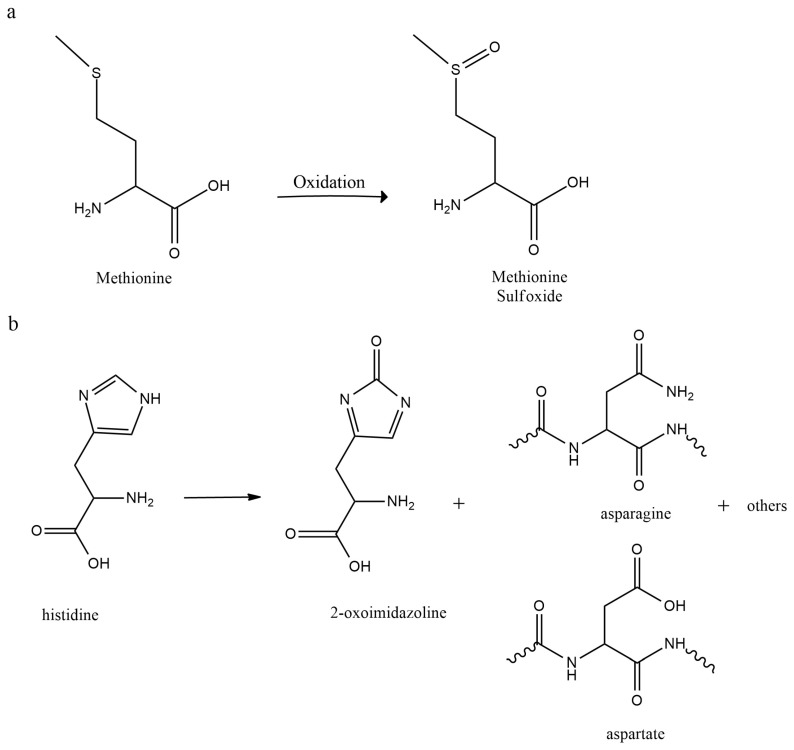
Oxidation reactions of Met and His: (**a**) oxidation by hydrogen peroxide to methionine sulfoxide in an acidic solution and (**b**) conversion of histidine to 2-oxo-his, asparagine, and aspartate.

Peptide oxidation is a reaction that increases the electronegative atom content in a peptide molecule [86], where oxygen or halogens are typically the electronegative heteroatoms [87]. Sulfur-containing residues such as Met and Cys are particularly susceptible to oxidation because sulfur atoms are highly reactive and can easily lose electrons, forming sulfur radicals when exposed to reactive oxygen species (ROS). Aromatic residues such as His, Trp, and Tyr are also prone to oxidation because the aromatic rings in these residues contain multiple carbon-carbon double bonds that are easily oxidized by various ROS (see Figure 3) [88].

Oxidation can be induced by contaminating oxidants, trace amounts of catalytic redox-active metals, and light exposure. Moreover, peptide oxidation may be affected by pH, temperature, and buffer composition [8]. Deprotonation of the mercapto group of Cys [89] and the phenoxy group of Tyr accelerates oxidation of these residues [90]. Deprotonation of the imidazole side chain of His favors metal binding and, potentially, oxidation [91].

#### 2.2.1. Autoxidation

Frequently, the oxidative degradation of pharmaceuticals is referred to as “autoxidation”. However, the term “autoxidation” denotes “the spontaneous oxidation in an air of a substance not requiring catalysis” [92]. Hence, if peptides were to autoxidize, this would require the reaction of amino acids with molecular oxygen. In general, the reaction of “closed-shell” (i.e., non-radical) organic substances with oxygen is relatively slow [93], and it is unlikely that autoxidation contributes significantly to peptide oxidation except, perhaps, to the oxidation of the mercapto group of Cys under the condition that chain oxidation is possible. Conditions for the chain oxidation reaction of dithiols (i.e., dithiothreitol) have been defined by radiation chemical techniques [94].

#### 2.2.2. Metal Induced Oxidation

Metal ion-catalyzed oxidation for peptides refers to the process by which metal ions can promote the oxidation of specific amino acid residues in peptides. This process usually requires the presence of a redox-active transition metal such as Fe^2+^ and Cu^2+^ that can undergo redox cycling reactions and produce ROS. In metal ion-catalyzed oxidation, metal ions act as catalysts, accelerating the conversion of hydrogen peroxide, superoxide anion radical, and hydroxyl radical. These hydroxyl radicals can then react with amino acid residues in peptides, causing degradation. Specifically, metal ion-catalyzed oxidation can cause oxidative damage to amino acid residues such as histidine (His), cysteine (Cys), and methionine (Met) [95]. Metal ion-catalyzed oxidation frequently implies a site-specific reaction catalyzed by transition metals complexed by metal-binding sites. Hence, metal ion-catalyzed oxidation frequently does not target the most solvent-accessible amino acids, but rather amino acids which are part of or are located close to metal ion-binding sites [96]. It was reported that the oxidation of hPTH (1–34) by ferrous ethylenediaminetetraacetic acid (EDTA)/H_2_O_2_, found that this system can cause oxidation of the methionine residue at position 8 (Met 8) and the histidine residue at position 9 (His 9) (1–34). The study found that the oxidation of Met 8 and His 9 in hPTH (1–34) resulted in the formation of sulfoxide and imidazole-5-aldehyde products, respectively. The oxidation of Met 8 was found to be highly selective, as this residue was oxidized much more rapidly than other methionine residues in the peptide. The oxidation of His 9 was also found to be highly selective, as other histidine residues in the peptide were not oxidized under these conditions. The study suggested that the oxidation of Met 8 and His 9 in hPTH (1–34) by ferrous EDTA/H_2_O_2_ may be relevant to the physiological and pathological roles of this peptide. For example, the oxidation of Met 8 may affect the biological activity of hPTH (1–34), as this residue is important for binding to the PTH receptor. The oxidation of His 9 may also affect the conformation of the peptide, as this residue is located near the N-terminus of the peptide and plays a role in stabilizing the peptide structure [97].

#### 2.2.3. Light-Induced Oxidation

Light-induced oxidation usually affects peptides that contain aromatic amino acid residues such as Trp, Tyr, and Phe, or a disulfide bond [46]. The mechanisms of light-induced oxidation are complex and not completely understood. While much emphasis has been placed on the primary photophysics and photochemistry of Trp, Tyr, Phe, and cystine, secondary reactions can induce the formation of a large variety of products [47]. The photo-irradiation of Trp can lead to photoionization as well as the formation of singlet oxygen. Photoionization is associated with the release of an electron, which can react with suitable electron acceptors such as oxygen (to yield superoxide) or disulfides (to yield thiolate and thiyl radical) [48]. Similar mechanisms have been reported for Tyr and Phe, though photoionization may be a biphotonic rather than monophotonic process. The biphotonic process is initiated by two-photon absorption, whereas the monophotonic process involves a single photon. Oxytocin was reported to be sensitive to U.V. light at pH 4.0–5.0 and 7.0–8.0 [49]. Recently, a series of papers have focused on near U.V. and visible light-induced photo-oxidation of peptides promoted by ligand-to-charge-transfer (LMCT) pathways of iron-buffer complexes [98,99,100]. These processes yield multiple reactive species and peptide oxidation products at relatively low light doses.

#### 2.2.4. Peroxide Oxidation

Peroxide can cause the oxidation of amino acid residues including Met [101], Cys [102], and His [103], as well as the formation of hydroperoxides on amino acids and polypeptides during oxidative stress, which can potentially lead to biological damage. Accidentally, peroxide may be present in formulations due to the inclusion of surfactants or other excipients. For example, some surfactants, such as polysorbate 20 and polysorbate 80, can produce peroxide [104]. Therefore, surfactants or co-solvents such as polyethylene glycol usually have certain specifications related to the levels of peroxides.

### 2.3. β-Elimination

A disulfide bond of a peptide can undergo β-elimination leading to C-S cleavage, resulting in perthiolate/perthiol and dehydroalanine. It is frequently observed when materials are subjected to high temperatures in conjunction with a high pH environment. Cys and Ser-containing peptides undergo β-elimination at alkaline pH [105,106]. Even at neutral pH, when cystine-containing peptides are heated at 100 °C, they initially form perthiol and then convert to free thiols [107]. sCT degrades through β-elimination at the disulfide bridge between the Cys residues at positions 1 and 7. It has also been reported that the insertion of an additional sulfur forms a trisulfide and tetrasulfide bridge because of a β-elimination reaction [108]. It has also been observed in oxytocin after exposure to heat stress at an alkaline pH [26]

### 2.4. Disulfide Exchange

Disulfide exchange reactions can occur in peptides, leading to disulfide scrambling and contributing to forming dimers and larger aggregates. An investigation on the degradation of sCT recognized dimeric products generated through disulfide exchange reactions. However, dimers linked to disulfides can go through further disulfide reactions, ultimately regenerating monomers of sCT [108]. In an acidic aqueous solution, disulfide interchange can continue through the formation of sulfonium ions [109]. When disulfide bonds are subjected to hydrolysis, sulfenic acid intermediates are formed, which can further react with other cysteine residues or with water to produce sulfonium ions. These ions can then undergo disulfide interchange reactions, leading to the formation of new disulfide bonds between cysteine residues. There have been several studies conducted on disulfide exchange reactions and the significance of disulfide bridges in maintaining peptide stability. Several investigations have highlighted the importance of disulfide bonds for peptide stability and the impact of disulfide exchange reactions on peptide conformation and function. By developing strategies to stabilize disulfide bonds and prevent disulfide exchange reactions, researchers can improve the stability and bioactivity of peptides for use as therapeutic agents [105,108,109,110,111,112].

### 2.5. Dimerization, Aggregation, and Precipitation

Apart from intermolecular disulfide bond formation, peptides can dimerize/oligomerize via a series of oxidative reactions [28,113,114]. Some of these processes may even lead to larger aggregates. In addition, stress conditions, such as freezing, heating, or agitation, may induce aggregation. Aggregates can form through covalent bonds; such as dityrosine, ester, disulfide, or amide linkages; or electrostatic interactions or non-covalent bonds that occur through hydrophobic interactions. However, during sample preparation, relatively weak non-covalent bonds may be disrupted again, leading to incorrect results [115].

The formation of aggregates on peptides is not limited to a single pathway [32]. Instead, multiple mechanisms can occur concurrently, leading to the formation of both soluble and insoluble aggregates [116]. Aggregation occurs when peptides interact with each other to form larger, multi-molecular species, which can have altered conformation, solubility, and biological activity. At higher concentrations, peptides are more likely to interact with each other due to increased intermolecular forces, resulting in faster aggregation. As aggregation proceeds, the peptides can become more insoluble and eventually precipitate out of solution [117]. In addition to precipitation, higher concentrations of peptides have been reported to form gel-like aggregates. Calcitonin, deterelix, leuprolide, and β-amyloid peptide are examples of peptides that are capable of forming gel-like aggregates under certain conditions [118]. Gel-like aggregates form because the structure shifts from an α-helix or β-turn structure to a β-sheet structure. As a result, they have strength, elasticity, and plasticity that can maintain their shape.

## 3. Strategies to Optimize Peptide Stability in Aqueous Formulations

Peptides are inherently unstable in aqueous solutions due to their susceptibility to degradation, aggregation, and other types of physical and chemical instability. To improve the stability of peptides in aqueous solutions, various strategies have been developed, including the use of buffers, organic solvents, specific metal ions, and air exclusion/oxygen removal (see Table 4). To optimize the utilization of formulation strategies for stable injectable peptide development, a deep understanding of peptide structure, physicochemical properties, and degradation pathways is required.

**Figure 4:**
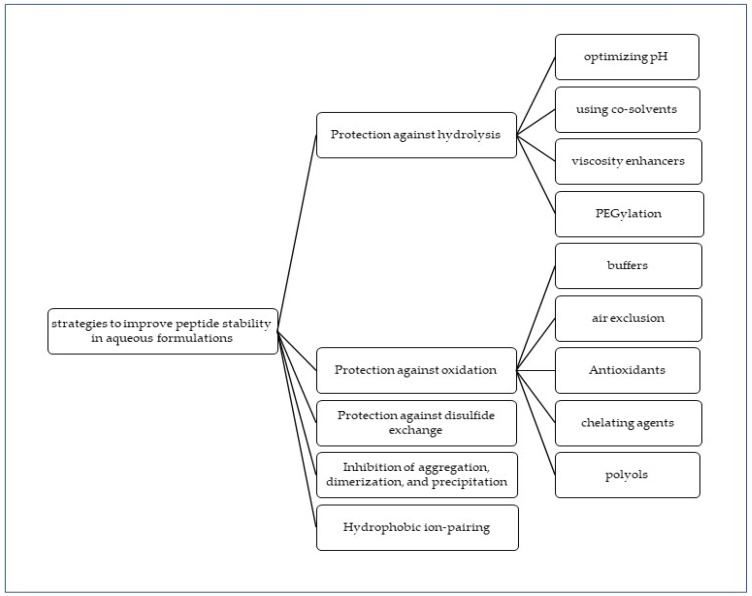
Known strategies that can be used individually or in combination to improve peptide stability in aqueous formulations.

Peptides differ from proteins in that they lack tertiary and quaternary structures due to their shorter length, and therefore, the side chains of amino acid residues are predominantly exposed to solvents and solutes. This exposes hydrophobic residues such as Trp, Tyr, and Phe to aqueous environments, leading to degradation. By analyzing a peptide’s amino acid sequence, scientists can gain insight into its susceptibility to degradation via various pathways, including oxidation and deamidation, and identify potential enzymatic cleavage sites. Secondary structures, such as alpha-helices and beta-sheets, can also contribute to peptide aggregation and precipitation. To improve peptide stability, appropriate formulation strategies can be designed, such as substituting susceptible amino acids or utilizing stabilizing agents, based on an understanding of the amino acid sequence and degradation susceptibility. Figure 4 summarizes various strategies for enhancing peptide stability in aqueous formulations.

### 3.1. Protection against Hydrolysis

#### 3.1.1. pH Optimization

Maintaining the stability of peptides in aqueous solutions often requires controlling the pH. Using buffers is a common strategy to prevent degradation. To ensure patient comfort during injection, the acceptable pH range for intravenous administration is typically between 3 and 10.5, while for other routes of administration, the range may be narrower [134,135]. Hence, at the start of formulation development, it is essential to evaluate the pH-dependent degradation of a peptide in the pH range 3–10, adjusted with various types of buffers at different concentrations [136,137]. To minimize deamidation, formulations should preferably be in a pH range between 3 and 5 [76,138,139]. Oxytocin, for example, exhibits the highest stability at pH 4.5 [26].

The vast majority of organic compounds can go about as radical scavengers [139], particularly hydroxyl radical [140]. However, most organic compounds may not prevent more selective oxidants such as peroxyl radicals. Few buffers can tie directly to peptides, thereby increasing their conformational stability [38,141,142]. For instance, citric acid buffers have been reported to increase oxytocin stability. Even though citric acid reacts with oxytocin forming N-cytril oxytocin, it was observed that fewer degradation products were formed in the presence of divalent metal ions [39]. It was reported that the carboxylate group of aspartate buffer has the ability to neutralize the positive charge of the N-terminus of Cys [142], thereby facilitating interactions with Zn^2+^, resulting in protection against dimerization of the disulfide bridge [38,142]. Octastatin, a somatostatin analog, was found to degrade greater in citrate or phosphate-containing buffers than in glutamate or aspartate buffers at pH 4.0 [69]. Additionally, octreotide has been reported to have better stability in acetate buffers at pH 4.0 [143].

#### 3.1.2. The Use of Co-Solvents

Using co-solvents can improve peptide stability in an aqueous solution. An aqueous solution’s dielectric constant can be decreased by adding organic solvent, resulting in a significantly lower rate of isomerization and deamidation [83]. The lower the dielectric constant of the solvent, the easier two differently charged (+, −) molecules contact each other. It is also possible that the reduced water content affects the rate of deamidation. E.g., peptide deamidation in an aqueous solution can be slowed down by the addition of glycerol [144,145], propylene glycol [138,146], or ethanol [147]. A formulation in an aqueous citrate buffer at pH 5.75 consisting of ethanol and propylene glycol increases the stability of eptifibatide [122].

#### 3.1.3. Viscosity Enhancement

In liquid preparations, the rate of chemical reactions decreases as the solution viscosity increases. It has been reported that various compositions of a combination of glycerol and polyvinylpyrrolidone (PVP) can have an impact on the rate of deamidation of peptides. This study has proven that PVP at high concentrations can decrease the rate of Asn deamidation of a hexapeptide. However, it is still uncertain whether the decrease in deamidation rate was caused by a reduction in the dielectric constant of the solution by glycerol, increased viscosity, or a combination of both [144,148]. PVP without glycerol has also been successfully used to inhibit the rate of the Asn-hexapeptide deamidation in aqueous solutions. PVP can interact with the peptide through hydrogen bonding, electrostatic interactions, and hydrophobic interactions, and act as a physical barrier between the peptide and the water molecules [117,146,149]. Effects of stabilization increase with increasing concentration and molecular weight, hence the viscosity [144,150]. A polymeric surfactant, such as Pluronic^®^ F68, may have a double effect as a polymeric surfactant and viscosity enhancer. Its surface acts as an interface protectant and has been used to improve ceftazidime stability in parenteral formulations [120]. Furthermore, high concentrations of Poloxamer 407 slowed down the deamidation rate of Asn residue in a model peptide Val-Tyr-Pro-Asn-Gly-Ala in an aqueous solution. The reduction of degradation rate was ascribed to the formation of an aqueous gel-altering solution conformation of a peptide and to the salting-out effects of the Poloxamer 407 [125].

#### 3.1.4. PEGylation

The covalent linkage of water-soluble polymers such as polyethylene glycol (P.E.G.) to peptides (PEGylation) can offer many advantages, including extended shelf life, improved water solubility, and stability under stressed conditions [124]. P.E.G. conjugation targeted site-specific amino acids, including lysine (Lys), Cys, arginine (Arg), and Tyr, which can increase the molecular size of the peptide. PEGylation has been reported to increase oxytocin stability at high temperatures [124], extending the biological activities of Human Pancreatic Polypeptide (hPP) [151] and minimizing side effects of antimicrobial peptides LyeTx I-b [152]. Although it is rare, it has been found that patients can develop allergic reactions to P.E.G. [153,154]. Therefore, PEGylated formula, although it has advantages, still needs further development.

### 3.2. Protection against Oxidation

The mechanisms of oxidation may vary depending on several factors, such as pH, the presence of oxygen, metal ions, and/or light, which can lead to damaging effects. Nevertheless, it is possible to mitigate such effects by adjusting the pH, eliminating oxygen from the solution, modifying the primary and secondary packaging to prevent light exposure, and employing antioxidants or metal chelators in the formulation. Waterman et al. have developed a comprehensive guideline for the use of excipients to enhance the oxidative stability of actives, which includes recommended concentrations [155].

#### 3.2.1. Buffers

Buffer solutions can be used to help prevent peptide oxidation, particularly for peptides that contain Cys, Met, Trp, Tyr, and His side chains. The choice of buffer can have a significant impact on peptide stability, and several factors should be considered when selecting a buffer for a specific peptide. Cys and Met residues are generally the most susceptible to oxidation in peptides due to the presence of sulfur atoms in their side chains. Cysteine can be oxidized to cysteine sulfinic acid or cysteine sulfonic acid, while Methionine can form methionine sulfoxide or methionine sulfone. Cys, Tyr, and His can be more susceptible to oxidation at neutral and alkaline pH due to deprotonation of their side chains. In contrast, an acidic environment (pH < 5) may reduce the susceptibility to oxidation of Cys, Tyr, and His residues by protonating their side chains and decreasing their reactivity with reactive oxygen species [33]. Compared to Cys and His residues, however, oxidation of Met and Trp residues are less affected by pH. The oxidation of Met can be promoted at a very low, clinically irrelevant pH (below 2) [156].

#### 3.2.2. Air Exclusion

Special handling is required during processing to minimize the exposure of peptide drugs to oxygen and other oxidizing agents that can cause damage. The manufacturing steps should be done by purging the container with an inert gas such as argon, helium, or nitrogen before adding and mixing the peptides. The filling steps must be performed using a pre-filled gas-tight container with inert gas. Additionally, it is crucial to handle the peptides gently and avoid agitation or shear stress, as these can cause structural damage and increase their susceptibility to oxidation [157]. The effect of temperature on oxygen solubility also needs to be considered during processing because dissolved oxygen concentrations at low temperatures is higher in an aqueous solution [158].

#### 3.2.3. Antioxidants

Antioxidants protect peptides from oxidation during processing and storage by scavenging reactive oxygen species. The choice of the appropriate antioxidant will depend on several factors, such as the specific amino acid residues present in the peptide, the formulation, and the intended use of the peptide. It is crucial to ensure that the selected antioxidant is compatible with the peptide and does not interfere with its activity or stability [33]. For instance, sodium bisulfite can be problematic for specific peptides because it is a nucleophile, meaning it can react with disulfide bonds, potentially leading to the formation of peptide aggregates and loss of activity [159]. Additionally, bisulfite can be oxidized to form the radical sulfite anion, which reacts with oxygen to generate peroxyl radicals—potent oxidizing agents that can damage peptides. Bisulfite can also react with amino acids containing thiol groups, such as cysteine, forming disulfides that can impact stability [160]. Similarly, adding ascorbic acid to peptide solutions contaminated with trace metal ions may not necessarily protect the peptide against oxidative modification. In some cases, it may accelerate the oxidation process, as shown by ascorbic acid’s tendency to advance Met oxidation in small model peptides and form Met sulfoxide [161,162]. Met is a sulfur-containing amino acid that can act as a sacrificial antioxidant, rapidly oxidizing to form methionine sulfoxide in response to numerous reactive oxygen species [163].

#### 3.2.4. Chelating Agents

Chelating agents protect peptides from oxidation by sequestering metal ions that can act as catalysts for the reaction. In pharmaceutical liquid formulations, various chelating agents are commonly used, including ethylenediaminetetraacetic acid (EDTA), diethylenetriaminepentaacetic acid (DTPA), desferal, ethylenediamine-di-o-hydroxyphenyl ace-tic acid (EDDHA), inositol hexaphosphate, tris(hydroxymethyl)aminomethane (TRIS), tartaric, and citric acid. EDTA is a versatile chelating agent that can bind to various metal ions, including copper, iron, and calcium. DTPA is particularly effective in binding to calcium and zinc ions. Desferal is used to treat iron overload, while EDDHA is used primarily for its ability to bind to iron ions in agricultural applications. Inositol hexaphosphate, a naturally occurring chelating agent, is particularly effective in binding to iron ions and has potential use in cancer treatment. TRIS is a buffering agent with some chelating properties, while tartaric acid is commonly used in the food industry to improve the stability and solubility of products. Citric acid is another chelating agent widely used in the food industry and pharmaceutical formulations, and is particularly effective in binding to calcium ions and other metal ions such as iron and copper. The choice of chelating agent will depend on the specific metal ions present in the formulation and the desired outcome [146].

However, adding specific chelating agents may accelerate the oxidation process of peptide molecules. Under certain conditions, chelating agents can bind to trace metal ions and form complexes with higher redox potential than metal ions alone, increasing the rate of oxidative reactions. In addition, some chelating agents may also generate free radicals during their interactions with metal ions, which can promote the oxidative degradation of peptides [33,164]. In an illustrative example, adding EDTA to a small peptide containing Met and His changed oxidation selectivity, targeting His instead of Met only [165]. It is also important to understand that EDTA/metal complexes are not always inert to oxidants. For example, [Fe(II)EDTA]2- reacts rapidly with hydrogen peroxide [166], ultimately generating both complexed and free hydroxyl radicals, which can attack all amino acids in a given peptide. Recently, triethylenetetramine was shown to be more effective than EDTA in protecting proteins (insulin and a monoclonal IgG) against Cu^2+^-mediated oxidation [167]; however, it has not yet been tested for peptides.

#### 3.2.5. Polyols

Polyols have been shown to protect therapeutic peptides from oxidation by scavenging reactive oxygen species (ROS). The hydroxyl groups in polyols can donate hydrogen atoms to ROS, inhibiting the ability to oxidize peptides. Some polyols commonly used to protect therapeutic peptides from oxidation are maltose, sucrose, trehalose, raffinose, and mannitol. For instance, mannitol has been shown to protect Met-containing peptides from iron-catalyzed oxidation [168], and sucrose has been shown to reduce the oxidation rate of both human brain natriuretic hormones (hBNP) and human parathyroid hormones (hPTH) [129]. A high concentration of sucrose (as much as 1 M) has also been shown to increase the stability of hPTH and hBNP in liquid formulations. The sucrose stabilizing effect was predominately due to the retardation of aggregation, oxidation, and deamidation of the peptides [129]. Sucrose induced small conformation changes in the hPTH structure, preferentially excluding oxygen from the peptide surface and maintaining the native conformation of hBNP, leading to a more compact peptide structure [129].

### 3.3. Protection against Disulfide Exchange Reaction

It was found that formulating octreotide in 10 to 60 mM glycine with pharmaceutically acceptable salts and HCl to adjust the pH values in a range of 3.0 and 4.2 are effective in protecting the cleavage of its disulfide bridge [169]. Octastatin was also reported to be more stable in a glutamate buffer at pH 4.0 rather than in an acetate or citrate buffer [69]. The combination-specific buffers with divalent metal ions may protect peptide drugs against disulfide exchange. We have reported that combining zinc, calcium, and magnesium ions with dicarboxylic and tricarboxylic acids can improve the stability of oxytocin [39,141].

### 3.4. Inhibition of Aggregation, Dimerization, and Precipitation

Aggregation and dimerization of peptides can occur through the formation of covalent bonds such as disulfide bridges and dityrosine, or non-covalent interactions such as hydrophobic forces. These aggregates can exist in both soluble and insoluble forms. Optimizing the pH and ionic strength of the solution can stabilize peptide aggregation in aqueous solutions [170,171,172,173,174]. For instance, the use of citrate buffers and divalent metal ions have been shown to inhibit oxytocin dimerization mediated by cysteine.

Another strategy to minimize a peptide’s aggregation is using extremolytes. Extremolytes are small organic molecules generated by extremophilic microorganisms that can safeguard biological macromolecules and cells from damage caused by external stresses including high temperatures and high salt concentrations [175]. Several studies have reported that extremolytes can stabilize peptides by creating solute hydrate clusters that are excluded from the peptide hydrate shell because of the repulsive interactions between the extremolytes and the peptide backbone. Water accumulation near the peptide area arranges the peptide into a more compact structure with a reduced surface area [176,177,178,179,180]. Some examples of extremolytes that have been shown to stabilize peptides in solution include polyol derivatives: ectoine and hydroxyectoine [181], trehalose [182], betaine [183], amino acids (e.g., proline), and mannosylglycerate [184]. Studies have shown that mannosylglycerate can stabilize β-amyloid peptides by inhibiting their aggregation [185].

Furthermore, sucrose, amino acids, and surfactants (polysorbate 20 and 80) [32] can be used with preferential exclusion to prevent dimerization. Polyethylene glycol (PEG) has been shown to reduce peptide aggregation by creating a steric barrier around the peptide molecule, preventing the close contact between peptide molecules that is required for aggregation [54,117,146,149,186,187,188]. The stabilizing effect increases with increasing concentration and molecular weight and, therefore, with increasing viscosity [83,144,150]. Peptide aggregation can also be reduced by dicarboxylic amino acids such as aspartic acid (Asp) and glutamic acid (Glu) through their ability to act as hydrogen bond donors and acceptors, enabling them to participate in intermolecular hydrogen bonding with other amino acid residues in the peptide, which in turn prevents the formation of insoluble aggregates [31,189]. Arg, Gly, and Lys have also been reported to prevent aggregation at neutral pH, since, at this condition, the positive charges of the amino groups electrostatically hinder the intermolecular interaction of a peptide [31,189,190,191]. Polysorbates can reduce agitation-induced aggregation of peptides, presumably due to a decreased exposure of peptide molecules to air/liquid interface [192,193]. However, some reports suggest that these surfactants are less effective in reducing thermally-induced aggregation [192,193,194,195].

### 3.5. Hydrophobic Ion-Pairing (HIP)

Hydrophobic ion-pairing (HIP) is a current strategy used to enhance the stability of therapeutic peptides in aqueous solutions. This technique involves the formation of ion pairs between a hydrophobic counterion and a positively charged amino acid residue in the peptide, typically His, Lys, or Arg. This interaction effectively shields the charged groups from the surrounding solvent, reducing their exposure to water and potential hydrolysis [196,197,198].

One advantage of the HIP strategy is that it does not involve chemical modification of the peptide, which can affect its biological activity, and may lead to undesirable side effects. In addition, HIP is a simple and effective approach that has been shown to enhance the stability of a wide range of therapeutic peptides, including glucagon-like peptide-1 (GLP-1) and somatostatin analogs [199,200].

Jörgensen et al. introduce biodegradable arginine-based steroid-surfactants as cationic green agents for hydrophobic ion-pairing, demonstrating their effectiveness in stabilizing model peptides under various stress conditions, such as high temperature and low pH. In addition, the study highlights the use of sustainable, biodegradable materials in designing the ion-pairing agents as an eco-friendlier approach to drug delivery [201].

## 4. Conclusions

Compared to proteins, peptides are generally more susceptible to degradation in aqueous solutions due to their smaller size and less complex structure. Unlike proteins, peptides do not have a well-defined 3D structure and are less flexible but more ordered, owing to fewer interactions and the potential to adopt multiple conformations. This exposes most amino acid residues’ side chains to solvent, allowing maximum contact with solvents. Hydrophobic side groups of amino acids such as Trp, Tyr, and Phe in peptides are buried inside their structure and, therefore, not or less exposed to the aqueous environment. By understanding the peptide structure and degradation pathways, one can develop strategies for adequate stabilization.

To ensure the stability and efficacy of injectable peptides, unique formulations and preservation methods may be necessary. Designing a therapeutic peptide formulation begins with knowing the amino acid sequence to predict potential degradation pathways and characteristics of therapeutic peptides. pH plays a vital role in peptide stability, so selecting a buffer to maintain the desired pH is a common strategy to reduce degradation rates in an aqueous solution. Buffer solutions in pH between 3–5 diminish deamidation and oxidation and provide disulfide bridge protection against exchange reactions. Some peptides may require excipients such as amino acids, sugars, or buffer systems to reduce degradation. Co-solvents, air exclusion, viscosity enhancement, bivalent cations, PEGylation, and polyol excipients are practical strategies to enhance peptides’ stability in solution. Additionally, aqueous injection peptides are often stored at low temperatures and protected from light to minimize degradation. Hydrophobic ion pairing (HIP) is an effective method for enhancing the stability of peptides in aqueous solutions. The technique involves introducing a hydrophobic counter-ion that forms a stable ion pair with the peptide’s positively charged amino acid residues. Biodegradable materials such as arginine-based steroid-surfactants can be used as green cationic agents for HIP, offering a more sustainable approach to drug delivery. It is essential to assess the ideal formulation for preserving the stability of a particular peptide against degradation for every distinct stress it may encounter.

Existing strategies for improving peptide stability and delivery have limitations and challenges that need to be addressed. One limitation is that these methods may not work for all peptides, or may not be effective under certain stress conditions. Additionally, some excipients used for stabilizing peptides may have adverse effects, such as inducing immune responses or altering the pharmacokinetics of the peptide. Another challenge is the delivery of peptides to the target site. Peptides can be rapidly degraded in the bloodstream, limiting their bioavailability and therapeutic efficacy. Therefore, alternative delivery methods, such as oral, transdermal, or nanocarriers, have been explored to improve peptide delivery. Improving peptide stability and delivery remains a critical challenge in peptide-based therapeutics. Therefore, future research should focus on developing innovative and effective strategies to overcome these limitations and challenges.

Future research directions include developing new strategies for improving peptide stability and delivery, such as using stabilizing agents specifically designed for a particular peptide, developing delivery systems that can protect peptides from degradation in the bloodstream, and improving the design of nanocarriers for more efficient peptide delivery. Furthermore, exploring new drug delivery routes, such as the oral route, may be necessary since oral administration of peptides is often more patient-friendly and cost-effective than injectable delivery. Another area of research is developing new formulations that can both withstand harsh conditions in the digestive system and effectively transport peptides to the target site.

## Figures and Tables

**Table 1:** Approved peptide drugs in the United States, Europe, and Japan from 2020.

Peptide	Year of Approval	Indications	Dosage Form	Administration Route
Setmelanotide	2020	Chronic weight management	Liq. Inj	Subcutaneous
64Cu-Dotatate	2020	Radiopharmaceutical	Liq. Inj	Intravenous
Vosoritide	2021	Pediatric bone growth	Powder for Inj	Subcutaneous
Difelikefalin	2021	Pruritus with chronic kidney disease	Liq, Inj	Intravenous
Melphalan flufenamide	2021	Relapsed or refractory multiple myeloma	Powder for Inj	Intravenous
Voclosporin	2021	Lupus nephritis	Capsule	Oral
Piflufolastat F 18	2021	Radiopharmaceutical	Liq. Inj	Intravenous
Pegcetacoplan	2021	Paroxysmal noctural hemoglobinuria	Liq. Inj	Subcutaneous
Dasiglucagon	2021	Hypoglycemia	Liq. Inj	Subcutaneous
Tirzepatide	2022	Type 2 diabetes	Liq. Inj	Subcutaneous
Terlipressin	2022	Hepatorenal syndrome	Powder for Inj	Intravenous

**Table 2:** Peptide drug candidates undergoing clinical development [3].

Peptide	Target Receptor	Indication(s) for Investigation	Clinical Trial Phase
TT-232 BPI-3016 NBI-6024 Many more	Somatostatin GLP-1 TCR	Renal cell adenocarcinoma Type 2 diabetes Type 1 diabetes	I
Angiotensin 1–7 Bombesin Cenderitide Deslorelin Gastric inhibitory polypeptide MK-3207 Olcegepant Pancreatic polypeptide Peptide YY (3–36) Somatoprim Thyrotropin	AT 2 Bombesin NPRA and NPRB GnRH GIPr CGRP CGRP Neuropeptide Y4 Neuropeptide Y2 Somatostatin TSH	Miscellaneous Peripheral Blood Cell Abnormalities Prostate cancer Heart failure Puberty; precocious Type 2 diabetes Migraine Migraine disorders Type 1 diabetes Metabolic disease; obesity Acromegaly Benign nontoxic and toxic goiter; goiter; nodular	II
Albusomatropin Anamorelin G17DT Insulin peglispro Selepressin Somapacitan Taspoglutide Tirzepatide Ularitide Vapreotide Vosoritide Zoptarelin doxorubicin	GHR GHSRCCK-2 IR V1A GHR GLP-1 GIP and GLP-1 NPR Somatostatin 2 and 5 NPR-B LHRH	Growth hormone deficiency Cachexia; lung cancer non-small cell cancer Various forms of cancer Type 1 and 2 diabetes Shock, septic Adult growth hormone deficiency Type 2 diabetes Type 2 diabetes Decompensated heart failure Gastri varices; esophageal haemorrhage; portal hypertension; esophageal varices Achondroplasia Endometrial cancer; prostate cancer	III
Avexitide Calcitonin gene-related peptide Corticorelin Leptin Thymalfasin	GLP-1 CGRP-R CRF-1 LEP-R TLR	Hypoglycemia Migraine Brain neoplasms; brain swelling Obesity; lipodystrophy Liver cirrhosis, sepsis	IV

**Table 3:** Degradation pathways of peptide in aqueous solution, critical parameters, and amino acid residue(s) involved.

Degradation Pathway	Critical Parameters	The Amino Acid Residue(s) Involved	References
Chemical Instability			
Hydrolysis	pH Temperature	Trp Ser Asn-Pro Asn-Tyr	[26,35,36,37]
Deamidation	pH Temperature	Asn Gln	[35,36,38,39,40,41,42]
β-elimination	Thermal stress pH	Cys-Cys	[35,37,43,44]
Oxidation	pH Temperature Oxygen	Trp Met Cys Tyr His	[36,37,39,44,45]
Light-induced oxidation	Light	Trp	[46,47,48,49]
Metal induced oxidation	Metal ions (copper, iron)	His Cys Arg Pro Met	[50,51]
Disulfide exchange	pH Oxygen Metal ions	Cys-Cys	[38,52]
Physical Instability			
Adsorption	Container	His Arg	[53]
Aggregation	Stress condition Concentration pH	Cys-Cys Tyr-Tyr	[39,40,42,43,54,55,56,57,58,59,60]

**Table 4:** Peptide therapeutic degradation pathways and possible stabilization strategies.

Peptide	Number of A.A.	Degradation Pathway	Stabilization Strategy	A.A. Residue(s) Involved	References
Thyrotropin-releasing hormones (T.R.H.)	3	Hydrolysis	pH 6.5	Glu	[119]
Ceftazidime	5	Hydrolysis	Pluronic^®^ F68 pH 4.5–6.5	Glu	[120,121]
Eptifibatide	6	Hydrolysis Isomerization Deamidation Oxidation Dimerization	pH 5.7 Co-solvent 0.025 M citrate buffer	Asp Cys-Cys	[122]
Octreotide	8	Hydrolysis Disulfide exchange	Air exclusion Buffer pH close to 4	Tyr Trp	[69,123]
Oxytocin	9	Oxidation β-elimination Deamidation Hydrolysis Dimerization Light-induced oxidation	Antioxidant pH 4.5 Acetate/Citrate/Aspartate buffer Divalent metal ions Protect from lightPEGylation Cyclization	Tyr Cys Cys-Cys	[35,38,39,43,44,45,124]
Desmopressin	9	Oxidation Deamidation Disulfide exchange β-elimination Racemization	Surfactants Polyols Buffer Divalent metal ions Phosphate buffer (pH 4.5–5.5)	Asn Gln Cys Tyr	[32,52,125]
Leuprolide	10	Hydrolysis Isomerization β-elimination Oxidation Aggregation	pH 3–5 Acetate buffer Co-solvent (DMSO)	Ser Trp	[126]
Goserelin	10	Hydrolysis Debutylation Epimerization	pH 3–5 Acetate buffer Co-solvent	Ser	[62]
Gonadorelin	10	Hydrolysis Deamidation Epimerization	pH 3–5 Acetate buffer Co-solvent	Ser	[62,64]
Triptorelin	10	Hydrolysis Deamidation Epimerization	pH 3–5 Acetate buffer Co-solvent	Ser	[62,64]
Somatostatin and analogs	14	Hydrolysis Disulfide exchange	pH 4–5 Acetate buffer NaCl	Trp-Tyr Trp-Lys Cys-Cys	[69,123]
Liraglutide	30	Aggregation Oligomerization	pH > 6.9	-	[55,127]
Salmon Calcitonin	32	Deamidation Dimerization Aggregation Hydrolysis Disulfide exchange	pH 3–4 Citrate buffer Phosphate buffer	Asn Gln Cys-Cys Cys-Ser	[57,108,128]
Human Brain Natriuretic Peptide [hBNP(1–32)]	32	Aggregation Deamidation Oxidation	Sucrose Air exclusion	Met Asn	[129]
Human Parathyroid Hormone [hPTH(1–34)]	34	Oxidation Deamidation Aggregation Cleavage Asp residue	Sucrose Co-solvent Air exclusion	Asp Asn	[49,115]
Adenocortico-tropin hormone (ACTH)	39	Hydrolysis Deamidation	pH 3.0–5.0 Acetate buffer	Asn Met	[71,130]
Amyloid-β (Aβ) peptides	36–43	Metal-catalyzed oxidation Deamidation Dimerization Aggregation	Chelating agents Polyols	His Cys Arg Pro Met	[131,132,133]
Exenatide	39	Aggregation Oxidation Deamidation	pH 4.5 Polyols	Gly Met Asp Trp	[42]

## Data Availability

Not applicable.
